# Osteoporosis in Rett Syndrome: A Study on Normal Values

**DOI:** 10.1100/tsw.2006.266

**Published:** 2006-12-15

**Authors:** Lilit Zysman, Meir Lotan, Bruria Ben-Zeev

**Affiliations:** ^1^ National Evaluation Team, Israel Rett Syndrome Center Chaim Sheba Medical Center, Tel HaShomer, Ramat Gan, Israel; ^2^ Department of Physical Therapy, Academic College of Judea and Samaria, Ariel, Israel

**Keywords:** Rett syndrome, osteoporosis, calcium levels, bone density, ultrasound, Israel

## Abstract

Osteoporosis is the reduction of calcium density in bones, usually evident in postmenopausal females, yet the tendency for osteoporosis can also be identified at a young age, especially in patients with chronic diseases, disabilities, and on chronic anticonvalsant treatment. Individuals with Rett syndrome (RS) have been found to show signs of osteoporosis at a young age. This condition may cause pathological fractures, inflict pain, and seriously damage mobility. In such cases, the quality of life of the individual and her primary caretakers will be severely hampered. This article reviews the current knowledge of the phenomenon and suggests some clinical directions for the individual with RS who shows signs of osteoporosis. The article also presents novel findings from a screening test of bone strength in 35 individuals with RS at different ages using the Sunlight Omnisense 7000P ultrasound apparatus. The primary results from this investigation showed a strong and significant positive correlation between calcium intake and bone strength (*p* < 0.0001) as well as bone density Z values (*p* < 0.005). The occurrence and frequency of fractures were found connected with reduced bone strength in measurements of both the radius (*p* < 0.0001) and the tibia (*p* < 0.004) as well as with negative bone strength Z values (*p* = 0.03). Other findings specified within the content of the article support the implementation of a comprehensive antiosteoporotic preventive management for this population.

## INTRODUCTION

Osteoporosis (osteo = bone, porosis = pores) was defined at a 1993 consensus conference as "a systemic skeletal disease characterized by low bone mass and micro-architectural deterioration of bone tissue with a resulting increase in fragility and risk of fractures”[1]. The usual populations at risk for osteoporosis are women at menopause age, yet the tendency for osteoporosis can also be identified at a young age[1] and the tendency for osteoporosis at adulthood is known now to be associated with the lack of achievement of maximal bone mass at a young age[1]. Lately it was established that children suffering from chronic disease were also a population at risk for osteoporosis[2]. Evidence suggests that 10% of children diagnosed with chronic disease might develop osteoporosis at a young age (ages 3–18 years) due to immobility and minimal exposure to sunlight[3]. Fractures due to osteoporosis are a major cause of long-term dysfunction and even death[4], especially for individuals with disability[5,6]. Bone mass density (BMD), a proxy for bone strength, was found to be reduced in children with disabilities compared with their healthy peers[7,8,9], which makes them prone to fractures of the long bones, occurring with minimal trauma[5,6]. These findings also apply to children with Rett syndrome (RS)[10].

## OSTEOPOROSIS IN RETT SYNDROME

The general risk factors for developing osteoporosis are:

1. Women and men of advanced age
2. Slim body build
3. Smoking
4. Menstrual irregularities
5. High caffeine consumption
6. High consumption of alcoholic drinks
7. A family history of osteoporosis
8. Low calcium nutrition
9. High salt and protein consumption
10. A sedentary life style
11. An early menstrual termination
12. Women who have not had child birth
13. The use of certain drugs such as anticonvulsive medication

Risk factors 2, 4, 8, 10, 12, and 13 are highly characteristic of RS. Besides belonging to the chronic disease group, persons with RS are exposed to the following risk factors: They do not exercise (especially the nonambulatory ones), 30–90% of them are expected to develop epilepsy and will need the use of anticonvulsive medication[11,12,13,14,15], and many of them show menstrual irregularities[16].

When considering the risk of developing fractures due to osteoporosis, it appears that individuals with RS are again disadvantaged. In his article, Heaney[17] mentions falls, lack of soft tissue padding, inappropriate postural reactions, lack of bone strength, and poor nutrition as major contributors to fractures and these factors are common among individuals with RS.

Actually, the fact that individuals with RS are at risk of developing osteoporosis was established by Haas and associates[10]. This group of researchers compared mineral bone density, mineral bone content, and mineral density of the spine of 20 young females with RS with those of 11 females with CP (cerebral palsy) and 25 controls with no pathology and found significantly reduced measurements presented in individuals with RS. When differences in age and weight were adjusted, it was found that individuals with RS showed lower bone density at the level of osteopenia (see explanation below), in comparison to the other groups[10]. Their findings were supported by other groups as well[18,19]. Budden and Gunness[20] found similar results in a study of five children with RS. They suggested that slow bone creation at a young age in individuals with RS eventually causes low bone density in this population. They hypothesized that the influence of MECP2 (the gene responsible for RS) is not restricted to damaging brain tissues, but has a direct effect on bone development as well[20]. While the frequency of fractures has not been studied clinically in this population, it appears that there is a higher incidence in relation to individuals without RS[21].

## DIAGNOSIS OF OSTEOPOROSIS

In the absence of methods of measuring bone quality, the diagnosis of osteoporosis tends to be made on the basis of low bone density[22]. Since 75% of bone strength is determined by bone density, it is considered a good indicator for diagnosing osteoporosis. Degrees of osteoporosis are defined according to the ratio between the actual bone density found in a client and the expected bone density of his peer population (T-score).

- Normal BMD is defined as a T-score between +2.5 and –1.0 (i.e., the patient's BMD is between 2.5 standard deviations [SDs], above the young adult mean and one SD below the young adult mean).
- Osteopenia (low BMD) is associated with a T-score between –1.0 and –2.5, inclusive. Osteopenia is also a term used by radiologists to indicate that the bones on a plain X-ray film appear to be of decreased mineral content.
- Osteoporosis is defined as a T-score lower than –2.5[23].

It is estimated that a drop in 1 SD around the spine increases the chance for a fracture by 190%. A drop in 1 SD around the neck of the femur increases the chance for a fracture by 240%. A drop of 2.5 SD increases the chance for a fractures by 600–800% of that expected in the normal peer group.

## The Common Methods for Diagnosing Osteoporosis

The techniques for measuring bone may be divided into those that measure the central skeleton (spine, proximal femur, whole skeleton, etc.) and those that measure some part of the peripheral skeleton. Measurement of the central skeleton is most widely carried out using:

- Dual-energy X-ray absorptiometry (DEXA/DXA). This technique is based on low-level X-ray radiation. In this method, bone density is measured at two sites (the spine and the hips). There is level 1 evidence that DXA bone measurement is the most effective way to estimate fracture risk[24].
- Strength measurement in the peripheral skeleton that may be performed by quantitative ultrasound (QUS) is a widely reported technique. It is achieved by measuring the speed of soundwaves along the measured bone. There is evidence that QUS provides measurements of bone density that can be used to estimate risk with accuracy similar to DXA in elderly populations[25], as well as in children with CP[26].
- Other techniques for measuring peripheral bone density such as peripheral quantitative computerized tomography (pQCT), calcaneal and radial DXA, radiographic absorptiometry, have been found to differentiate between those with and those without prevalent fractures in postmenopausal Caucasian women[27].

## A RESEARCH PROJECT

The research population included 35 individuals with RS (see Table 1) with an age range between 2–39 years of age and a mean age: 12.8 (S.D. ± 8.8).

The equipment used in this study was an Omnisense 7000P, product of the Sunlight Company. The Omnisense 7000P device measures the speed of ultrasound waves propagating along the bone. The Z score calculations are done by comparing the speed of sound (SOS) with the mean SOS results of a young age, gender-matched, control population[28].

**TABLE 1**
**Details on Participants**

| Number of Participant | Age on Examination | Ambulation | | Phenotype | Mutation |
|---|---|---|---|---|---|
| | | Yes | No | | |
| 1 | 3 | | + | Classic | R255X |
| 2 | 5 | + | | Classic | T158M |
| 3 | 8 | + | + | classic | Macro_deletion |
| 4 | 3 | + | | Classic | Q244fs258x |
| 5 | 10 | | + | Classic | R255X |
| 6 | 15 | + | | Classic | 252258x |
| 7 | 11 | + | | Forme fruste | WT |
| 8 | 9 | | + | Classic | 4.2KB_Del |
| 9 | 13 | | + | Classic | R168X |
| 10 | 15 | | + | Classic | R133C |
| 11 | 4 | | + | Classic | 62+1delGT |
| 12 | 13 | + | | Angelman-like | A201V |
| 13 | 19 | + | | PSV | P152R |
| 14 | 15 | | + | Classic | R133C |
| 15 | 3 | | + | Classic | R306C |
| 16 | 7 | | + | Classic | R255X |
| 17 | 2 | | + | Classic | R255X |
| 18 | 10 | + | | Classic | R168X |
| 19 | 21 | + | | Classic | S134C |
| 20 | 13 | + | | Classic | 1303t400 del insertion P |
| 21 | 10 | + | | Classic | P360fs365X |
| 22 | 13 | + | | Classic | T158M |
| 23 | 20 | | + | Classic | R168X |
| 24 | 5 | + | | Classic | R106W |
| 25 | 17 | + | | PSV | L386-S401 Del 15 |
| 26 | 4 | + | | Classic | WT |
| 27 | 34 | + | | PSV | R133C |
| 28 | 12 | + | | Classic | R270X |
| 29 | 20 | | + | Classic | WT |
| 30 | 11 | | + | Classic | T158M |
| 31 | 19 | | + | Classic | R255X |
| 32 | 4 | + | | Classic | A2059fs266X |
| 33 | 39 | | + | Classic | T158M |
| 34 | 10 | | + | Classic | R168X |
| 35 | 3 | | + | Classic | L386fs431X |

Glossary: Wt = wild type, Del = deletion.

The research was held over a period of 2 years (January 2004 to December 2005) during routine annual and semi-annual follow-up examinations at the Israel Rett Syndrome Center. Every individual with RS in Israel visits the center on at least an annual visit. During those follow-ups, a standard bone strength measurement was conducted (by LZ). The test is not painful or intrusive and requires placement of an ultrasound transducer on the distal part of the tibia (when the participant is lying down) and the forearm (when the participant is seated at a table) according to a predetermined procedure. Data regarding the general disposition of each participant with RS were collected using a scale presented by Alison Kerr et al.[29] (0 = good, 1 = fair, 2 = worse).

## Statistical Analysis

ANOVA was employed through a general linear model using a statistical analysis system (SAS) program. Spearman correlation was employed on SAS to detect possible connections between different variables. Statistical significance was considered when $\alpha$ probabilities were found below 0.05 ($p < 0.05$).

## RESULTS

For the whole group of participants, the mean bone T-score values were found at –1.05 over the radius, placing the research population in the osteopenia range. On the other hand, the results from the tibia were found only slightly lower than the norm, putting them within the normal range. As expected and according with manufacturer's charts, a significant positive connection was found between measurement values and age ($p < 0.02$; b = 57; N = 27) Yet the bone strength of females with RS was found to deteriorate with age in comparison to normal values of females of the same age according to manufacturer data (see Fig. 1).

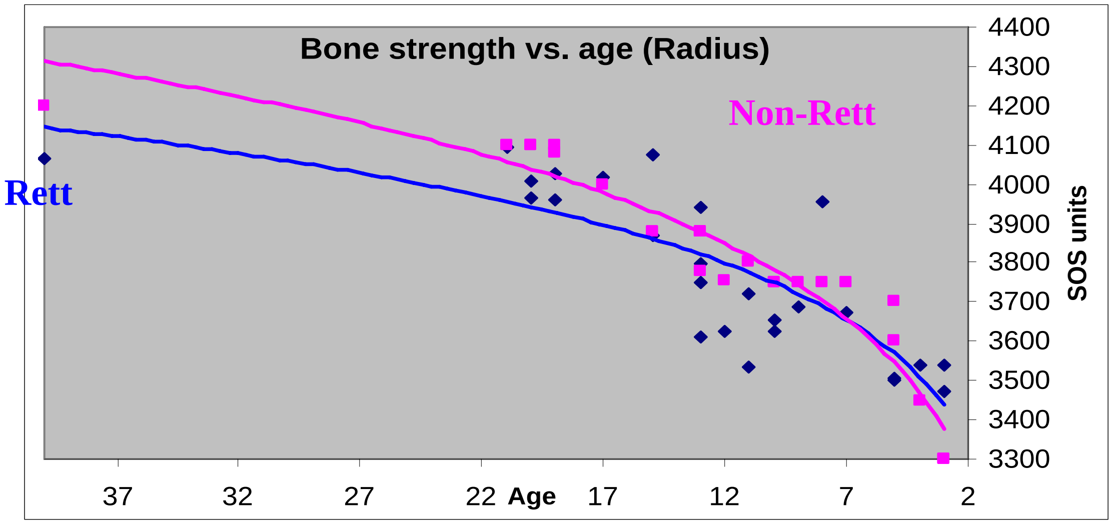

**FIGURE 1**. A comparison of bone strength values for Rett and non-Rett populations.

ANOVA was executed in order to detect possible connections between bone strength (SOS measurements) and bone Z values to all other parameters. The ANOVA procedure failed to detect any significant findings. Nevertheless, an effect bearing a trend towards statistical significance was noted on bone strength (at the tibia location), which correlated with mutation site. Individuals with MBD missense mutations (R133C; T158M) scored 206 SOS units higher than other mutations, with a value close to being significance at $p = 0.083$. This finding calls for further investigation with larger populations. No other significant findings could be drawn through the use of the ANOVA procedure.

Correlation in Spearman was executed with SAS software in order to detect primary correlative trends among different elements related to the present investigation. A list of dependent variables (bone strength at tibia and radius, bone Z values at tibia and radius sites) were searched for correlation with potentially influencing variables (height, age, birth year, weight, calcium intake, caloric intake, vitamin D consumption, mobility, fractures in the past, scoliosis, constipation, reflux, epilepsy, speech, level of breathing abnormality, manual functional level, head circumference, and genetic mutation type). Table 2 presents the significant findings.

**TABLE 2**
**Summary of Significant Findings**

| Spearman Correlation Coefficients Prob > \|r\| under H0: Rho=0 Number of Observations | | | | | |
|---|---|---|---|---|---|
| **Independent variables** | | **Dependent variables** | | | |
| | | **Radius** | **Tibia** | **Radius Z value** | **Tibia Z value** |
| Birth year | Correlation | -0.799 | -0.631 | -0.023 | 0.282 |
| | Statistical significance | <0.001** | 0.004** | 0.90 | 0.242 |
| | N* | N=27 | N=19 | N=29 | N=19 |
| Age | Correlation | 0.567 | 0.647 | 0.121 | -0.270 |
| | Statistical significance | 0.002** | 0.003** | 0.53 | 0.26 |
| | N* | N=27 | N=19 | N=29 | N=19 |
| Weight | Correlation | 0.685 | 0.443 | 0.118 | -0.158 |
| | Statistical significance | 0.0001** | 0.074 | 0.573 | 0.544 |
| | N* | N=23 | N=17 | N=25 | N=17 |
| Height | Correlation | 0.775 | 0.592 | 0.041 | -0.158 |
| | Statistical significance | <0.001** | 0.016* | 0.852 | 0.573 |
| | N* | N=21 | N=16 | N=23 | N=15 |
| **Linear regression describing possible predictors (when age, height and weight are neutralized)** | | | | | |
| Calcium | Correlation ($R^2$) | 0.67 | 0.40 | 0.23 | 0.14 |
| | Statistical significance | <0.001** | <0.001** | 0.020* | 0.9386 |
| | N* | N=22 | N=17 | N=24 | N=16 |
| Scoliosis | Correlation ($R^2$) | 0.67 | 0.52 | 0.12 | 0.12 |
| | Statistical significance | <0.001** | <0.001** | 0.051 | 0.958 |
| | N* | N=22 | N=17 | N=24 | N=16 |
| Epilepsy | Correlation ($R^2$) | -0.02818 | -0.28954 | 0.16078 | -0.31851 |
| | Statistical significance | 0.8913 | 0.2292 | 0.4137 | 0.1977 |
| | N* | N=26 | N=19 | N=28 | N=18 |
| Speech | Correlation ($R^2$) | -0.524 | -0.233 | -0.220 | 0.223 |
| | Statistical significance | 0.010* | 0.351 | 0.289 | 0.389 |
| | N* | N=23 | N=18 | N=25 | N=17 |
| Mobility | Correlation ($R^2$) | 0.69 | -0.25695 | 0.12 | 0.13 |
| | Statistical significance | <0.007** | 0.1957 | <0.025* | <0.89 |
| | N* | N=22 | N=27 | N=24 | N=16 |
| Breathing abnormalities | Correlation ($R^2$) | -0.97 | -0.52 | -0.27 | 0.14 |
| | Statistical significance | <0.001** | <0.001** | 0.008* | 0.95 |
| | N* | N=20 | N=16 | N=22 | N=15 |
| Fractures | Correlation ($R^2$) | -0.73 | -0.37 | 0.12 | 0.08 |
| | Statistical significance | <0.001** | <0.001** | 0.017* | 0.97 |
| | N* | N=19 | N=14 | N=21 | N=13 |
| Statistical significance was below 95-99%* above 99%**<br>N* - Not all participants were integrated in all calculations due to missing data<br>Shaded cells represent variables that were found statistically significant. | | | | | |

Calcium intake was found to be highly connected ($p < 0.0001$) with bone strength in measurements at both the tibia and radius when neutralizing age and weight parameters. Calcium was found connected with positive Z values in measurements at the radius ($p < 0.05$; b = –0.87; N = 5), but not the tibia; these results were not significant when neutralizing age and weight parameters (see Fig. 2).

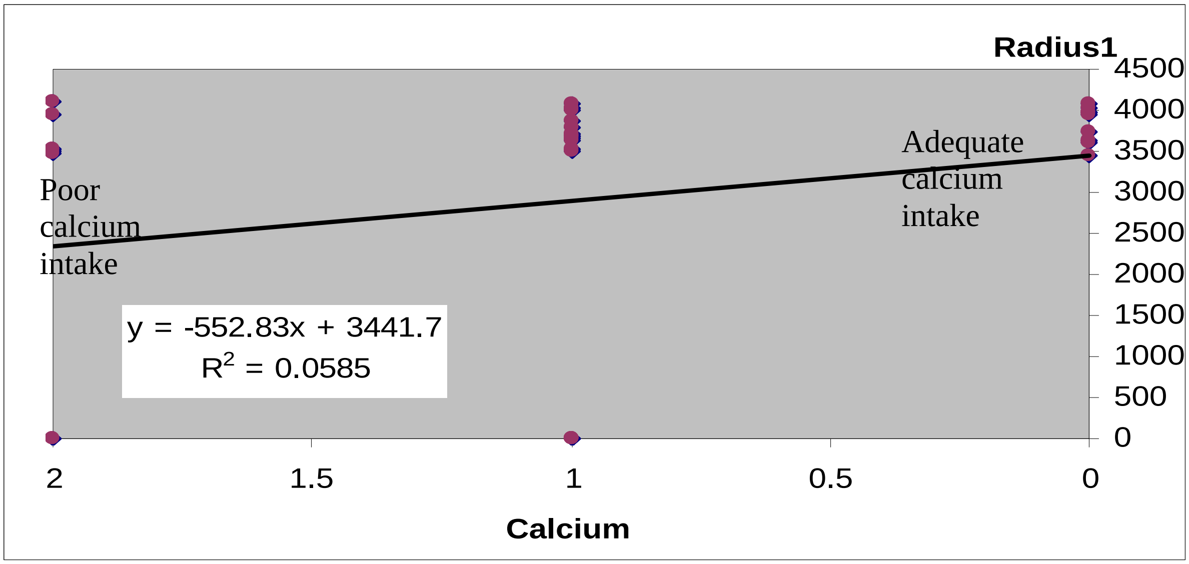

**FIGURE 2**. A correlation between calcium intake and bone strength.

For all participants (N = 35), low age (–0.08; $p = 0.075$) (see Fig. 3) and weight (–0.46; $p = 0.067$) variables (young participants) were closely connected (yet not statistically significant) with higher tibia Z values.

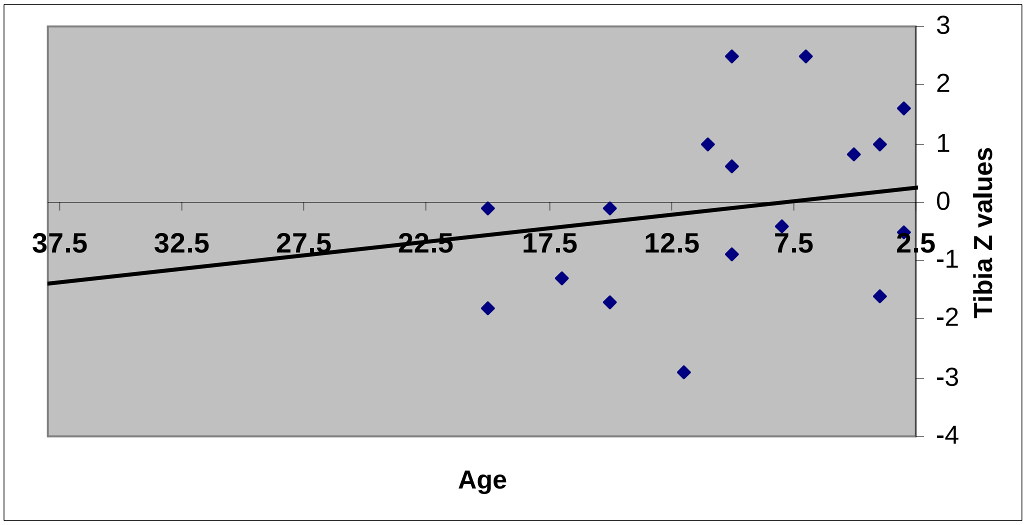

**FIGURE 3.** A connection between aging and low Z values.

For all participants (N = 35), high caloric intake was found connected with bone strength values in measurements of the radius ($p < 0.0007$) (see Fig. 4) and the tibia ($p < 0.005$) when neutralizing age height and weight.

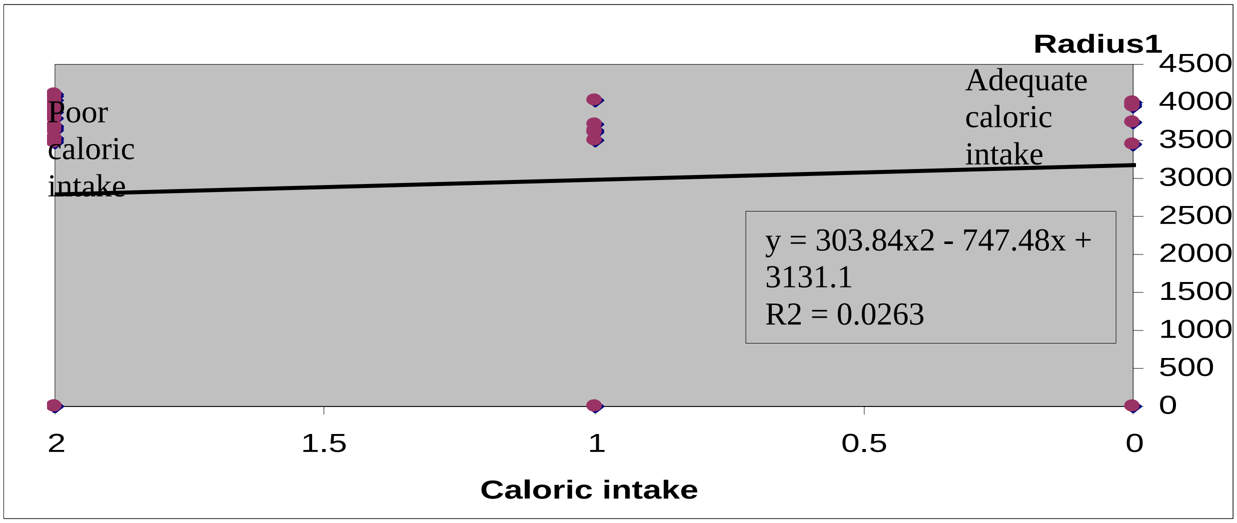

**FIGURE 4.** The connection between caloric intake and bone strength.

Fractures were found connected with reduced SOS units, representing bone strength values in measurements of the radius ($p < 0.0001$) and the tibia ($p < 0.004$) when neutralizing age height and weight. A strong and significant negative correlation was found between fractures and bone density Z values (N = 7; Correlation = –0.8; $p = 0.032$) (see Fig. 5).

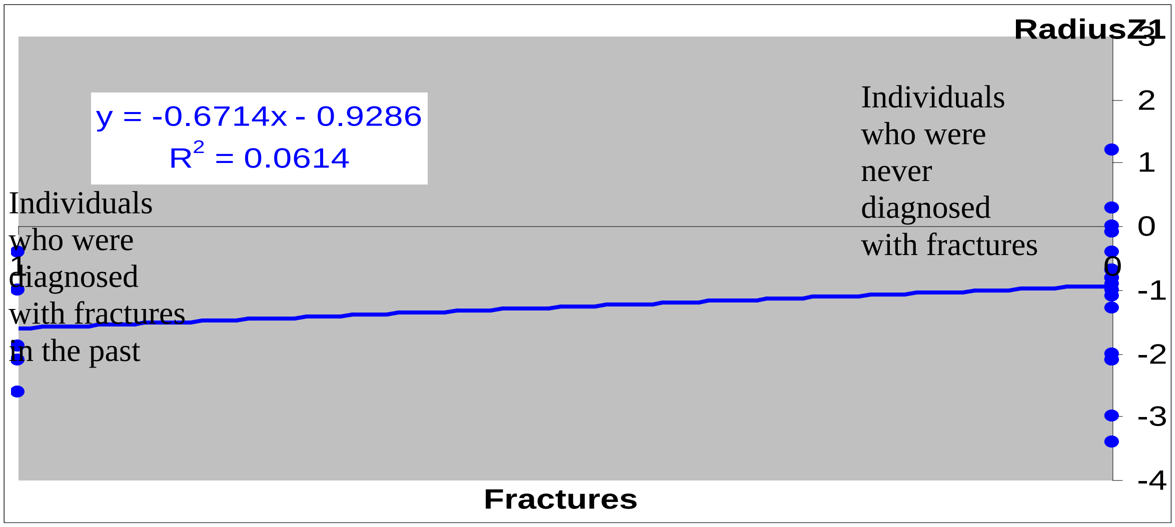

**FIGURE 5**. The connection between fractures and bone strength.

Mobility was found positively connected with bone strength values in measurements of the tibia ($p < 0.007$), but not the radius when neutralizing age height and weight (see Fig. 6).

Scoliosis was found negatively connected with bone strength values in measurements of the radius ($p < 0.001$) and tibia ($p < 0.013$) when neutralizing age height and weight (see Fig. 7).

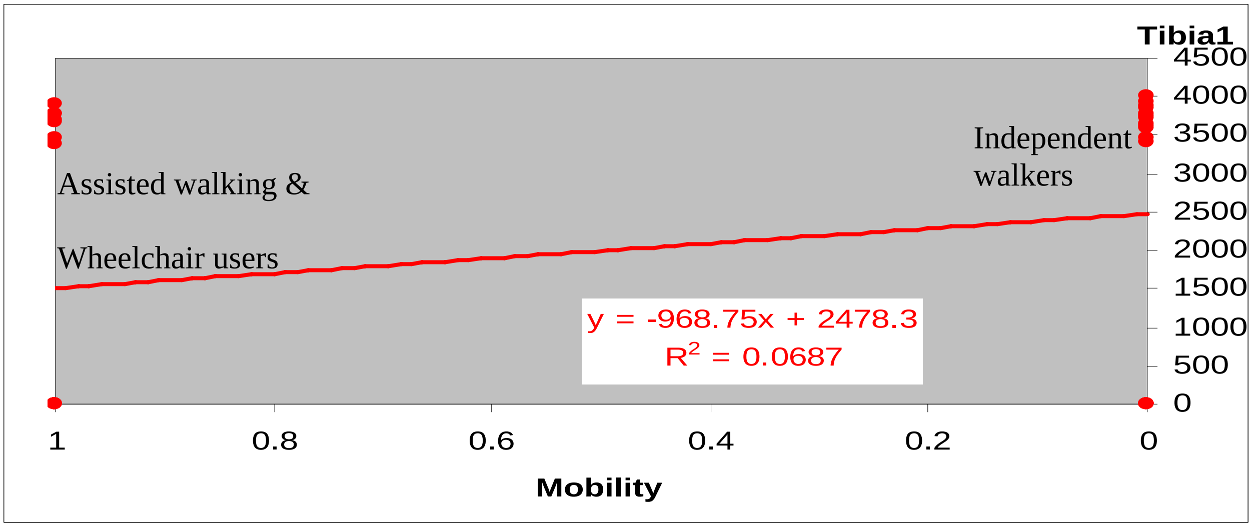

**FIGURE 6**. The connection between mobility and tibia bone strength.

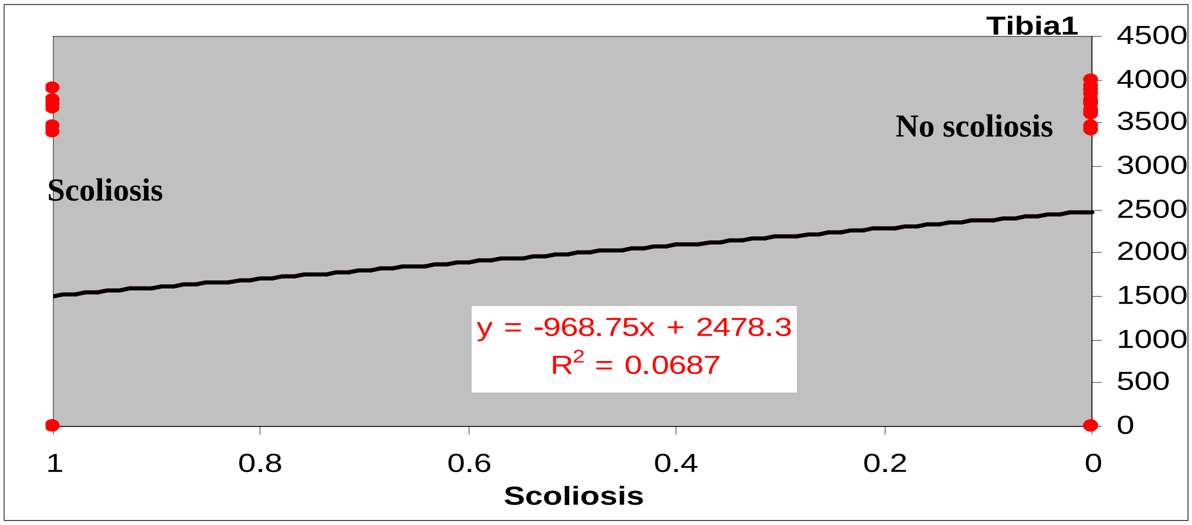

**FIGURE 7**. The connection between scoliosis and tibia bone strength.

Abnormal breathing was found negatively connected with bone strength values in measurements of the radius ($p < 0.001$) and tibia ($p < 0.003$) when neutralizing age height and weight. Epilepsy was found negatively connected with bone strength values in measurements of the radius ($p < 0.001$) and tibia ($p < 0.0003$) when neutralizing age height and weight.

## DISCUSSION

The information offered within the scope of the present article suggests some insights as to osteoporosis in individuals with RS. An overwhelming result was that out of 35 participants, only four showed positive Z values over the radius. Most of the findings support the results reported by others that individuals with RS show reduced levels of bone strength and bone density at the level of osteopenia in comparison to normative values. Yet our findings showed that bone strength levels revealed normative values at a young age and began to deteriorate towards the end of the first decade. These findings might collide with the hypothesis made by Budden and Gunness[20], who suggested that osteopenia in individuals with RS is connected to the primary activity of MECP2. Unless we assume that the inactivity of MECP2 in the skeletal system becomes apparent only at an age higher than 7 years (the age when RS bone strength is becoming inferior compared to normative findings). The most important finding was the fact that fractures in individuals with RS were found to correlate negatively with bone strength values as well as bone density Z values. This is an expected, yet alarming, result that necessitates proper intervention by caregivers.

The fact that calcium intake was found highly connected with bone strength as well as with positive Z values emphasizes the importance of calcium intake as preventive intervention for the development of osteoporosis in individuals with RS.

The fact that worse Z values were correlated with advanced age is not surprising and reiterates the importance of an antiosteoporotic intervention for this population from a young age.

The finding in the present research also supports the importance of proper high caloric nutrition as part of such an intervention.

The correlation found between mobility and tibia strength vs. reduced tibia strength and reduced mobility reiterates the importance of maintaining walking abilities in this population. These findings correspond with the findings of Cepollaro et al.[31]. It should be mentioned, however, that ambulatory status was not found connected with level of BMC (bone mineral content) by another group of researchers[19].

Abnormal breathing as well as epilepsy and scoliosis (all related to a more severe phenotype) were found to correlate with reduced bone strength. These findings suggest that applying an antiosteoporotic intervention plan is highly warranted when the individual with RS is showing worse phenotypic expression. The results of the present research urge the implementation of a comprehensive antiosteoporotic intervention from a young age for individuals with RS.

No difference was found in SOS measures between individuals with and without epilepsy at all ages. These findings are inconsistent with the findings of Cepollaro et al.[31], yet correspond with the findings of Motil et al.[19]. The lack of connection between bone strength and epilepsy might be explained by the small number of participants (N = 35) and the low number of individuals without epilepsy and with complete bone strength data (N = 4).

The present findings support the call of previous researchers to use ultrasound bone measurement equipment in screening tests designed to discriminate between healthy and osteopenic subjects[32,33,34].

## CONCLUSIONS

Osteoporosis is a multidisciplinary problem requiring proper diagnosis and organized intervention. In order to achieve good management, the appropriate experts should be consulted with a physician, a dietitian, and a physical therapist.
mechanical loading[36] has been found to enhance bone density and improved BMD in disabled children[37].

The basic steps of such an antiosteoporotic regime should include:

- A thorough evaluation of the individual with RS to sustain or eliminate a diagnosis of osteoporosis.
- In case the situation necessitates intervention, introduction of a comprehensive program is recommended.
  - Such a program should include three basic elements:
  - Medication — Such as estrogen hormonal treatment, kalcytonine hormone management (Miacalcic), Raloxifen (Evista), bifasfonates – or anabolic agents
  - Nutrition — Bone restoration will accrue if a positive nitrogen and mineral balance is sustained due to adequate intake of these nutrients. A well-balanced nutrition includes a wide range of provisions that may assist in keeping the strength and health of the bones in a constant, optimal state especially by keeping a diet that includes high caloric, calcium, and vitamin D intake.
- Exercise — Individuals who are more active present greater bone density and reduced chance for fractures than individuals that are inactive[35]. Individuals with RS should also be exposed to ample activity from a young age, and partake in an active life style in order for the individual to reach her maximal level of activity. For non-ambulatory individuals, even low-magnitude
